# Time-course of coherence in the human basal ganglia during voluntary movements

**DOI:** 10.1038/srep34930

**Published:** 2016-10-11

**Authors:** Omid Talakoub, Bogdan Neagu, Kaviraja Udupa, Eric Tsang, Robert Chen, Milos R. Popovic, Willy Wong

**Affiliations:** 1 Department of Electrical and Computer Engineering, University of Toronto, Canada; 2 Institute of Biomaterials and Biomedical Engineering, University of Toronto, Canada; 3 Toronto Western Research Institute – University Health Network, Canada; 4 Division of Neurology, Faculty of Medicine, University of Toronto, Canada; 5 Rehabilitation Engineering Laboratory, Toronto Rehabilitation Institute – University Health Network, Canada

## Abstract

We are interested in characterizing how brain networks interact and communicate with each other during voluntary movements. We recorded electrical activities from the globus pallidus pars interna (GPi), subthalamic nucleus (STN) and the motor cortex during voluntary wrist movements. Seven patients with dystonia and six patients with Parkinson’s disease underwent bilateral deep brain stimulation (DBS) electrode placement. Local field potentials from the DBS electrodes and scalp EEG from the electrodes placed over the motor cortices were recorded while the patients performed externally triggered and self-initiated movements. The coherence calculated between the motor cortex and STN or GPi was found to be coupled to its power in both the beta and the gamma bands. The association of coherence with power suggests that a coupling in neural activity between the basal ganglia and the motor cortex is required for the execution of voluntary movements. Finally, we propose a mathematical model involving coupled neural oscillators which provides a possible explanation for how inter-regional coupling takes place.

The brain consists of a number of distinct networks which are involved in the coordination of complex tasks. How is the coordination and communication between different networks achieved? For example, movement is regulated by several brain regions both in the cortical and the subcortical areas. The cortical region includes the primary motor cortex, while the corresponding subcortical regions include the basal ganglia as well as the thalamus^1^,^2^. A number of pathways have been identified connecting cortical to subcortical regions during motor control. The direct pathway (cortico-striato-internal pallido/nigral network) is thought to be responsible for initiating goal-directed behaviour such as executing voluntary movements. The indirect pathway is involved in movement inhibition and termination. Finally, the cortico-subthalamic hyperdirect pathway inhibits competing motor commands^2^,^3^,^4^. While the pathways themselves have been detailed extensively, little is known about the coordination of these regions.

One method to study these networks is to characterize movement in terms of electroencephalography (EEG) and local field potential (LFP) activities^1^,^5^,^6^,^7^,^8^,^9^,^10^,^11^,^12^,^13^,^14^,^15^,^16^,^17^,^18^,^19^. For example, it is well established that beta band activity in the cortex and in the basal ganglia are suppressed during movement^8^,^9^,^20^,^21^,^22^. By contrast, gamma rhythms increase during the movement phase in both the cortex and basal ganglia^5^,^22^. Interactions between the cortex and subcortical regions have been difficult to study in humans due to the relative inaccessibility of the basal ganglia^7^. Deep brain stimulation (DBS) is an accepted form of treatment for advanced dystonia and Parkinson disease (PD) and provides a unique opportunity to record electrical activities from the human basal ganglia in relationship to movement. We recorded the basal ganglia activity from patients when the DBS leads were temporarily externalized in the post-operative period while performing self-paced and externally cued tasks. Simultaneous recordings from deep brain electrodes and from the motor area allowed us to study detailed interactions between basal ganglia and cerebral cortex over time.

Earlier studies investigated the interactions between the basal ganglia and cortical regions in terms of the frequency content of neural activity but not how the interactions change over time^5^,^7^,^8^,^16^,^20^,^23^,^24^. More recently, a number of studies examined at time-dependent changes^9^,^21^,^22^,^25^ but none have looked at the time evolution of coherence in finer detail. To address this gap, we present a study here investigating the time-dependent coherence between basal ganglia and cortex, tracking coherence relative to both movement phase and LFP powers. Our results suggest a complex inter-play between different brain structures. A neural oscillator model is also proposed to help understand the mechanism of communication between different networks.

## Results

### ERS/ERD in the basal ganglia and in the motor cortex

**Figure 1:**
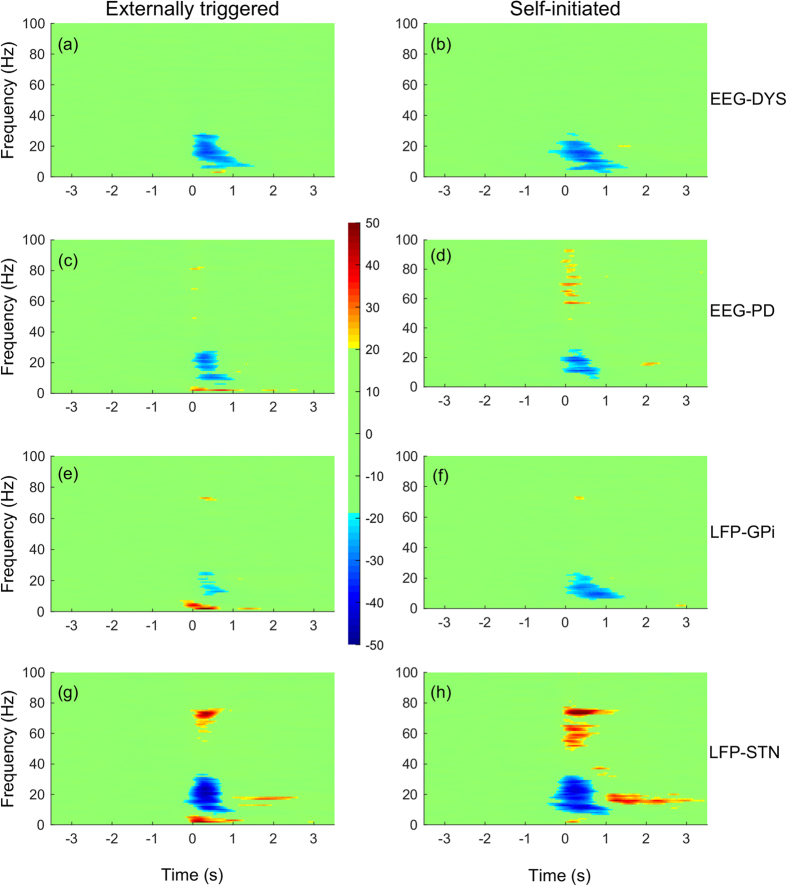
Percentage changes in power at the side contralateral to the moving wrist with respect to baseline. Baseline is defined as the average power between −4 and −3 seconds. Externally triggered movements are shown in first column, with self-initiated movements in second column. Trials are aligned such that the EMG onset is indicated by t = 0. (**a/b**) EEG recordings from dystonia patients (C3-Cz); (**c/d**) EEG recordings from Parkinson’s disease patients (C3-Cz); (**e/f**) LFP recordings from GPi of dystonia patients; (**g/h**) LFP recordings from STN of Parkinson’s disease patients.

**Figure 2:**
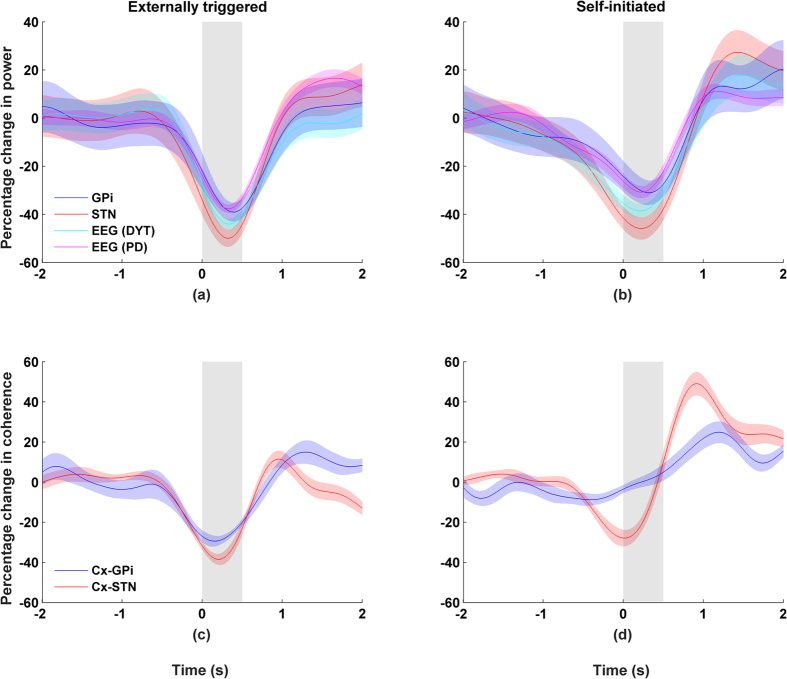
Beta band activity (20–30 Hz) recorded from the side contralateral to the moving wrist. Movement duration indicated by grey area and shaded areas indicate standard errors. Left column shows externally triggered task; right column self-initiated task. Percentage changes for beta band (20–30 Hz) showing (**a/b**) power and (**c/d**) coherence between cortex-GPi or cortex-STN.

**Figure 3:**
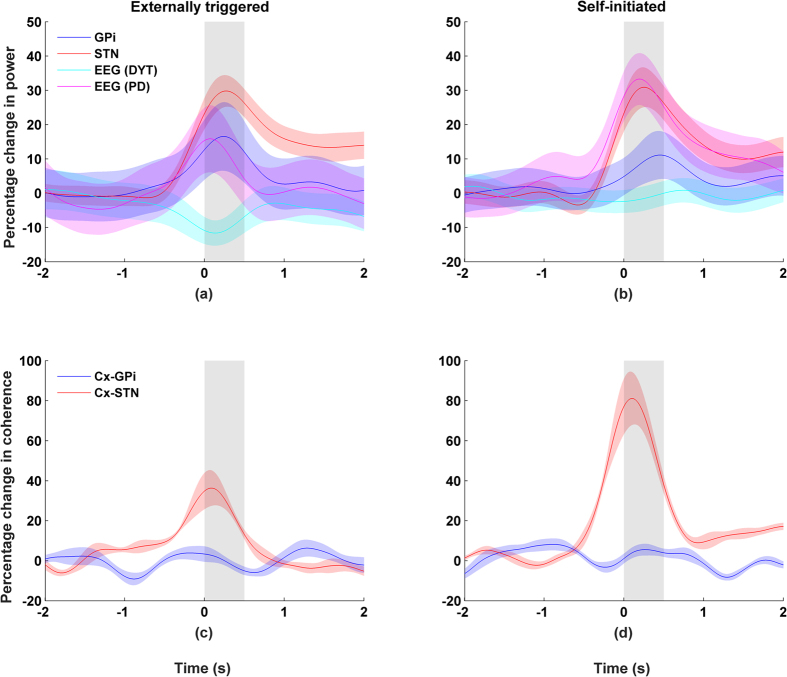
Gamma band activity (65–80 Hz) recorded from the side contralateral to the moving wrist. Movement duration indicated by grey area and shaded areas indicate standard errors. Left column shows externally triggered task; right column self-initiated task. Percentage changes for gamma band (65–80 Hz) showing (**a/b**) power and (**c/d**) coherence between cortex-GPi or cortex-STN.

**Figure 4:**
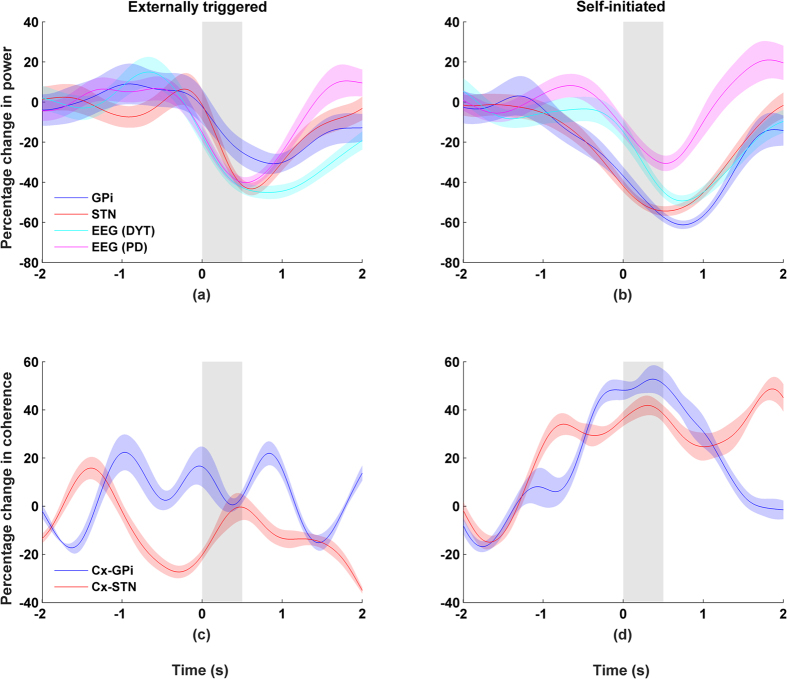
Alpha band activity (8–12 Hz) recorded from the side contralateral to the moving wrist. Movement duration indicated by grey area and shaded areas indicate standard errors. Left column shows externally triggered task; right column self-initiated task. Percentage changes for alpha band (8–12 Hz) showing (**a/b**) power and (**c/d**) coherence between cortex-GPi or cortex-STN.

Changes in the spectral density during wrist movement (i.e. event-related synchronization/desynchronization or ERS/ERD) in the subcortical and cortical regions are shown in Fig. 1. Time zero represents the onset of muscle activity as determined from EMG recordings of the extensor carpi radialis and flexor carpi radialis muscles. During the movement execution, which occurs approximately over the interval 0 and 0.5 second, there is an increase in low-frequency activity (<8 Hz) as well as gamma activity (65–80 Hz). Alpha-beta activity (8–30 Hz) on the other hand decreased during the course of the movement (p < 0.01, two-sample Kolmogorov-Smirnov test). The ERD in alpha-beta activity is observed bilaterally in both the motor cortex as well as in STN and GPi, lasting from two seconds before the movement onset to approximately two seconds after movement termination. Band-specific activities (α, β, and γ) are shown in Figs 2, 3 and 4 (a/b only).

More specifically, desynchronization in the beta band was found to be similar for self-initiated (r = 0.96, p < 0.01) and externally triggered (r = 0.92, p < 0.01) movements. Post-movement activity in the beta band shows a rapid rise, exceeding the baseline before gradually returning to baseline two seconds after movement termination. An ERS was found in the gamma band of both STN and GPi recordings for both externally triggered and self-initiated movements (p < 0.01, two-sample Kolmogorov-Smirnov test, Figs 1 and 3). However, the gamma activity was both larger in magnitude and bandwidth for STN than for GPi (Fig. 1). Based on these observations, we choose a common width for the gamma band (65–80 Hz), which encompassed gamma activity in both STN and GPi.

### Coherence between motor cortex and basal ganglia

Coherence was calculated between the motor cortex and STN, and between the motor cortex and the GPi under a variety of conditions. Cortical activities were recorded using a bipolar EEG montage (C3-Cz or C4-Cz). Subcortical activities were recorded using the DBS electrodes.

Figure 2 shows the time-course of coherence in relation to changes in power for high beta band activity (20–30 Hz). First, it can be observed that there is a high degree of synchrony at rest between cortical activity and STN, and cortical activity and GPi (p < 0.01, permutation test). Second, coherence was lowest during movement execution. These changes appeared to be correlated to the temporal changes in power (p < 0.01, Pearson correlation). These observations are consistent across self-initiated and externally-triggered trials. For ipsilateral recordings, the results were similar (Supplementary Figure S1).

While the results for coherence in the alpha band were derived from the same data used in the analysis of beta activity, we found no clear relationship in coherence between cortex-STN or cortex-GPi. Moreover, no significant differences in results were observed when coherence was calculated using ipsilateral versus contralateral recording sites (Fig. 4 and Supplementary Figure S3).

Coherence calculated in the gamma band show mixed results. First, we present the results obtained with contralateral recordings. We observed a strong increase in the coherence between the cortex and STN (Fig. 3), which was temporally aligned with the ERS in STN (r > 0.8, p < 0.01, Spearman correlation). No clear relationship was found between coherence and gamma power (r < 0.1, Spearman correlation) when the activities were recorded from the contralateral GPi and cortex. This may be related in part to the small magnitude in gamma ERS for GPi. For activities recorded from the ipsilateral side, we observed similar trends but they are less significant than that of the contralateral results (Supplementary Figure S2).

### Inter-hemispheric coherence

**Figure 5:**
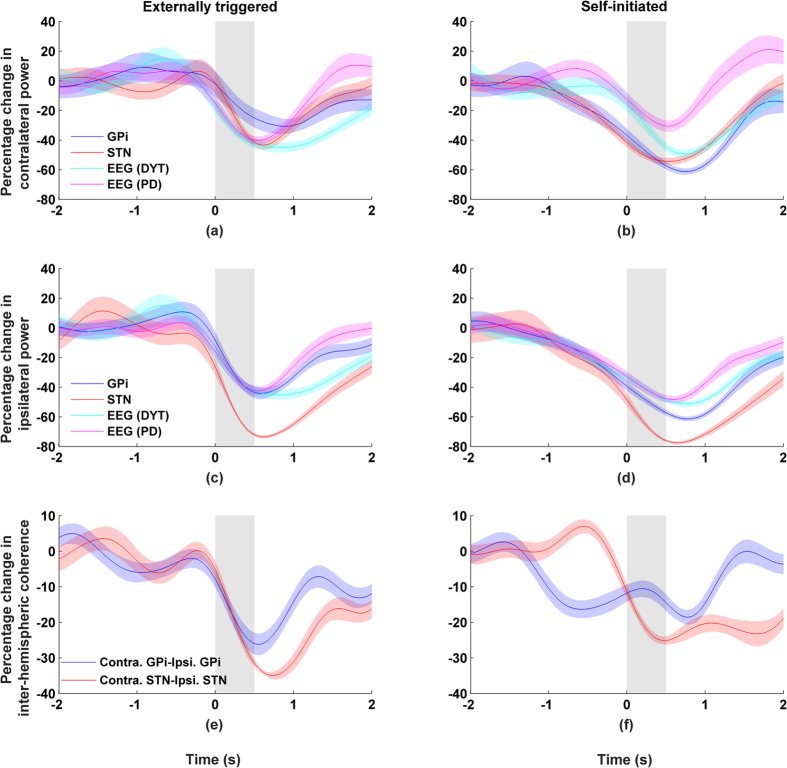
Activity from STN and GPi recorded from both the ipsilateral and the contralateral sides. Movement duration indicated by grey background and shaded areas indicate standard errors. Left column shows externally triggered task; right column self-initiated task. Percentage changes in alpha band (8–12 Hz) showing (**a–d**) power and (**e/f**) inter-hemispheric coherence between left-right GPi or left-right STN.

Despite a significant alpha ERD observed in both the ipsilateral and contralateral electrodes, no corresponding relationship was found for alpha coherence. Next, we examined coherence across GPi/STN of the left and right hemispheres. Around 5–10 Hz – and only in that frequency band – a very significant level of coherence was observed during idling state (i.e. before and after movement execution, p < 0.01, permutation test) as well as a drop during the course of wrist movement. These results are shown in Fig. 5 where the coherence in the alpha band is plotted together with changes in alpha power for both the GPi and STN. The coupling between power and coherence is significant (p < 0.01, Pearson correlation) and is similar to what was observed earlier in the beta and gamma bands for the basal ganglia and the cortex.

## Discussion

The interactions between the basal ganglia and the cortical regions have been investigated in terms of frequency content, but rarely in terms of its change over time^5^,^7^,^8^,^9^,^16^,^20^,^21^,^22^,^23^,^24^,^25^. Our study adds to the current body of knowledge by detailing cross-regional coupling of neural activity in detail finer than previously investigated. Time-dependent coherence between the basal ganglia and motor cortex was studied and tracked relative to movement and power. Our results show a complex inter-coupling between the sensorimotor cortex and basal ganglia in the high beta (20–30 Hz) and gamma (65–80 Hz) bands for both externally triggered and self-initiated movements. Beta band activity has a high degree of coherence during rest, but falls during the movement. The opposite was found for the gamma band. In both cases, coherence is essentially time-locked to the neural activity of the band. Similar results were observed for low beta band (12–20 Hz) but were not as dramatic. For alpha (8–12 Hz) and lower frequencies, coherence was not found to be correlated with power changes despite significant movement-related activities in these bands. Similar findings have been reported in earlier studies investigating the cortico-thalamic, cortico-GPi, and cortico-STN circuits^1^,^22^,^25^,^26^,^27^. These studies further confirm a lack of coherence in the cortico-basal ganglia-thalamic loop.

Changes in beta activity are associated with the processing of movement^8^,^9^,^20^,^21^,^22^. Moreover, abnormalities of these rhythms are thought to be cause of the symptoms associated with movement disorders like Parkinson’s disease^28^. Our observations on power and coherence are consistent with the hypothesis that beta activity plays a crucial role in movement termination and the idling of the motor system^6^,^9^,^21^,^29^,^30^,^31^. Similarly, an increase in gamma coupling during movement execution suggests that there is a communication channel linking the motor cortex with STN^1^,^5^,^7^. However, the coherence was not found to be significant between the GPi and the cortex. This mismatch may be due to differences in the pathways linking the various nuclei in the basal ganglia with the cortex. For example, a hyperdirect pathway links the cortex to the STN but no corresponding path can be found for cortex and GPi. The conduction time between the cortex and basal ganglia ranges between 1 to 3 ms for the hyperdirect pathway from cortex to the STN and about 20 ms for STN-GPi-thalamus-cortex pathway in humans^32^,^33^,^34^. Therefore, cortical and basal ganglia networks and their coherence can change rapidly in the same time frame as the movements themselves.

Gamma band and beta band activity are believed to arise from different mechanisms. Gamma activity is thought to reflect neuronal firing, while beta band activity reflects primarily synaptic inputs. Hence, post-synaptic potentiation is a possible binding mechanism across distant brain regions for beta oscillations. On the other hand, spike-to-spike coherence is expected to decrease as a function of frequency as fast dendritic currents are not reliably propagated through the synapse. Therefore, gamma band activity is considered to mainly reflect local neuronal firing and is not expected to bind distant regions^35^. A possible mechanism for cross-regional synchrony for gamma can be through long axons with short transmission delays. Short transmission delays result in synchronous action potentials across distal regions (for detailed review see ref. 36). Moreover, coherent local field potentials can also be the result of synchronous activity from upstream neurons with distributed axon terminals to downstream networks causing coherence between different regions. One such example is the feed forward network found in the visual and entorhinal-hippocampal systems^37^,^38^,^39^.

Inter-hemispheric coherence between left-right STN or left-right GPi was observed for alpha but not for beta or gamma despite high levels of activity in each band. The role of inter-hemispheric alpha is currently not well understood. Inter-hemispheric alpha may have a role in inhibiting unwanted movements and maintaining the idling state^40^,^41^. However, there are no direct anatomical connections between the nuclei on each side. Moreover, the lack of alpha coherence between BG and cortex suggests that inter-hemispheric coherence is mediated by other subcortical structures like the thalamus. The thalamus has been implicated as the generator and modulator of alpha activities^42^ and may in turn govern what messages get passed onto the cortex^43^,^44^. A drop in alpha coherence suggests that there is an opening of the communication channel between BG and cortex during movement. Thus, alpha activity can be modulator of beta/gamma activity – something that requires further investigation.

The results in this paper suggest that neural activity (ERS/ERD) is coupled to changes in coherence between the cortex and BG. This implies that coupling occurs when there is a sufficiently high level of neural activity. This is surprising for the simple reason that coupling (or coherence) measures the phase-locking of neural networks, and not its overall magnitude of neural activity. Coupling can occur even when neural activity is low, something that we do not find in our data. If there is noise in the system, however, it is entirely possible that the coherence is lost when the signals are weak relative to the noise (low signal-to-noise ratio). In this case coupling between coherence and power can be entirely artifactual. This has been reported previously as the so-called ‘power confound’ effect^45^ and can occur particularly when measurements are carried out using a common reference. Moreover volume conductance in the brain can similarly result in a high level of coherence between distant measurements.

We feel that the power confound effect and the effect of volume conductance cannot explain our findings for a number of reasons. First, our recordings are free of a common reference. Since we used bipolar recordings and calculated the difference between two closely spaced contacts, this greatly reduces (if not eliminates) the effect of a common reference as well as volume-conducted activity. Our results can therefore be thought to reflect activity that is local in origin^22^,^25^,^46^. Concerning the power confound effect, we observe clear cases where coherence remains high even when neural activity is low (e.g. see Fig. 3). Third, to investigate this further and more systematically, we plotted power vs. coherence for different time points during arm movement. As shown in the Supplementary Materials, single subject plots (as well as the grand average) show that power and coherence do not co-vary in any monotonic manner (Supplementary Figure S4). This is particularly striking at high activity levels where we would expect the clearest signs of any relationship – something that we do not observe.

What might be a possible purpose of coupling power with coherence? The “communication through coherence” hypothesis proposes that communication across neural networks takes place when the oscillatory activities between different brain regions are coherent^47^,^48^. Rhythmic patterns of excitability across communicating networks provide a temporal window for communication because the input-output channels of the networks are synchronized and open at the same times. Coherence thus enables a network to respond selectively to target inputs while ignoring other inputs^49^. A qualitative model is required to better understand the implications of coupling between coherence and power.

### Future Considerations

We present here some preliminary ideas on how coupled or phase-locked networks can be realized and detail a mathematical model that provides results qualitatively similar to what we observe in the coupling of coherency and power.

A neuron can be thought of as an oscillator with a natural frequency and phase. The frequency maps to the firing rate of the neuron, and the phase to the timing of the spike events. The Kuramoto model^50^ is one such model that has been widely studied and adopted (e.g. refs 51, 52, 53, 54 Networks of neurons can be modeled in terms of oscillators that are coupled together). One feature of the model is that the frequency of each oscillator can be changed or modified through pair-wise interactions with other oscillators. That is, through interaction and coupling, a network of oscillators can achieve phase or firing synchronization. The Kuramoto model has been applied to the study of the cortex^51^,^52^,^53^,^54^ as well as to the neurobiological basis of memory^55^.

**Figure 6:**
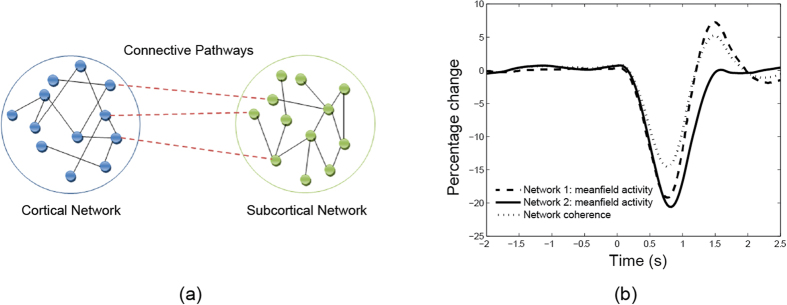
(**a**) Schematic illustration of the interaction between two neuronal populations. The neurons for each network are interconnected, with additional connections between the two networks. Such a network can provide a model describing the interaction between cortical and subcortical regions of the brain. (**b**) Simulated mean field activity (e.g. LFP) for two coupled networks of neurons. Coherence and power are high during idle period followed by a drop during movement.

The classic Kuramoto model assumes all-to-all connections between oscillators. Effectively this means that all the neurons are interconnected. Interaction between neurons can be modeled by the equation


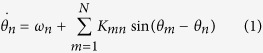


where 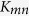 is strength of the interaction between a pair of oscillators or neurons, 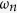 is the neuron’s natural frequency, and 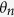 represents its phase. The sinusoidal function modulates the phase interaction between the neurons, limiting the magnitude of this interaction. We modified the traditional Kuramoto model in two ways. First, our model uses random connections so that each neuron is no longer connected to all other neurons. Second, since we are interested in studying the inter-relation between different networks (i.e. the cortical vs. subcortical networks), the model was constructed as two separate networks with additional connections linking these networks. Please see Fig. 6 for a schematic representation of the model.

In our preliminary simulations we have found the mean field activity of the network (i.e. the local field potential) to be coupled to the coherence between the two neuronal populations. Figure 6 shows the results from one simulation of a model with two networks, each of size 800 neurons. In each network, the neurons were interconnected with other neurons in the same network with 80% probability. For simplicity, the connections were set with identical coupling strengths. Finally the two networks were connected by 160 additional links.

In order to mimic the behaviour observed in our experiments, we varied the coupling strength of Network 1 resulting in an increase in the strength of the mean field. For the first simulation, coupling strength was increased linearly from 1.7 to 2.4 between time t = 0 s and 0.7 s, held constant and then decreased back to 1.7 between t = 0.8 and 1.5 s. We observe that the qualitative behaviour of the figure matches that of the results shown earlier for beta band activity between BG and cortex (see Fig. 2). Thus, the Kuramoto model, crude as it is, provides one possible explanation for why the coherence between networks is coupled to the activity in the networks. We can generalize these results further over a wide range of parameter values by demonstrating that the dynamical activity of the network converges to a limit cycle when the connectivity between the networks exceeds a threshold. Our findings are consistent with an earlier study by Mirollo and Strogatz^56^ where it was found that a high level of neural activity leads to an increase in coherence in population activity. We should emphasize that it would be desirable in future work to prove mathematically that such coupling can always be observed in such networks under a wide range of conditions. Our approach takes inspiration from existing works on modelling the network connectivity inside the basal ganglia (e.g. ref. 57); it is worth exploring the connectivity from a modelling viewpoint.

### Study Limitations

There are several limitations to our study. It is entirely possible that the findings in this paper are affected by the disease states of the individuals (i.e. Parkinson’s disease or dystonia) as it is not possible to record GPi and STN activity in normal subjects. The number of subjects in this study was also limited by the available pool of patients undergoing DBS treatment. Moreover the dataset collected is from a nonhomogeneous patient population Our results may also be affected by the microlesion effect^58^,^59^. However, since we have observed similar findings for both Parkinson’s disease and dystonia patients, our findings are likely to be physiological rather than specific to a particular disease condition.

## Conclusion

We have characterized the interaction between different brain networks during voluntary movements. Activity in the globus pallidus interna (GPi) and subthalamic nucleus (STN) was studied together with their interactions with the motor cortex during wrist movements. Both the power and the coherence in activity between the basal ganglia and motor cortex were found to be time-locked in the gamma and the beta bands. No such coupling was found for alpha activity although a high degree of inter-hemispheric coherence was observed. A model involving coupled neural oscillators was found to provide an explanation for the qualitative nature of our findings. Our observations suggest a complex interplay between the cortical and sub-cortical structures during movement execution and highlight possible neural mechanisms involved in motor coordination.

## Materials and Methods

### Patients

We studied seven patients with dystonia who had bilateral DBS electrodes in the GPi and six PD patients with bilateral DBS electrodes in the STN. Supplementary Table S1 shows their demographic and clinical details. The experiments were performed 1–3 days after DBS electrode implantation while the leads were externalized. The participants performed externally triggered and self-initiated brisk wrist extensions while sitting in a comfortable armchair. In the externally triggered paradigm, the participants faced a computer screen with dark background that turned green for 0.5 second at randomized intervals between 6 to 10 seconds. The participant was instructed to perform a brisk wrist extension when the green screen was shown. In the self-initiated paradigm, the participants performed self-initiated brisk wrist extensions every 10–15 seconds.

The participants provided written informed consent and the protocol was approved by the University Health Network Research Ethics Board. The experiments were performed in accordance with the approved protocol.

### Data recording and analysis

The participants were implanted with quadripolar DBS electrodes (Medtronic model 3387). The contacts were used to record GPi or STN local field potentials and were 1.27 mm in diameter and spaced 1.5 mm apart. EEG signals were recorded at Fp1, Fz, Cz, C3 and C4 (according to the 10–20 international system) and referenced to linked ears. The channels were sampled at 2.5 kHz and bandpass filtered between 0.05 Hz and 500 Hz. EMG was recorded from the extensor carpi radialis and flexor carpi radialis muscles to monitor wrist muscle activities. EMG signals were sampled at 2.5 kHz and bandpass filtered between 30 Hz and 500 Hz. SynAmp amplifiers (Neuroscan Laboratories, El Paso, Texas, USA) were used for all recordings.

The data was analyzed using MATLAB. First, monopolar DBS recordings were converted to bipolar recordings using difference between the adjacent contacts. This transformation allowed us to remove/suppress the common activities (including volume conductance and artifacts) while preserving the focal activities. The recordings were then divided into epochs consisting of 4 seconds before and 4 seconds after EMG onset. EMG onset was marked manually. Epochs with eye blink or other artifacts were excluded from the analysis. We obtain over 50 epochs from each participant for each movement condition. The epochs were then used to calculate the movement-related spectral changes and coherence.

### Movement-related synchronization/desynchronization

Changes in local field activity can be found either as a decrease in band power known as event-related desynchronization (ERD), or an increase in band power known as event-related synchronization (ERS). A spectrogram was used to represent the spectral density of the recordings over time. To obtain the spectrogram of each trial, the signal was divided into overlapping segments of 400 ms by applying a Hamming window. A Fourier transform was computed for the windowed signal resulting in a spectrum with resolution of 1 Hz. The window was then shifted by 10 ms (98% overlapping window), and the procedure was repeated until the end of the epoch was reached. The resulting spectrogram consists of a matrix in which each row represents the power spectrum of a windowed signal. The movement-related spectral changes were then calculated as percentage changes of the power with respect to a baseline, which we defined as the average power between 3 and 4 seconds prior to onset of muscle activity.

We analyzed the data recorded from the contacts selected for clinical purposes following programming of the stimulator. In some patients, the stimulator case was used as the cathode and a bipolar recording was obtained by subtracting the active contact with an adjacent contacts. The adjacent contact was chosen as the contact with maximum movement-related changes. The clinically-relevant contacts and the pairs chosen for our calculations are listed in Supplementary Table S1.

### Coherence between motor cortex and STN/GPi

Coherence was used to quantify the synchronous activity between two recording sites during voluntary movement. Coherence is defined mathematically as


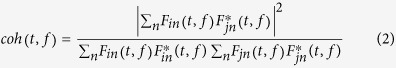


where *n* represents the trial number and 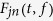 is the discrete Fourier transform of channel *j* at time *t* and frequency *f*. This measure of coherence quantifies phase-locking and similarity of spectral powers across repeated trials through a cross-correlation of spectral densities. Coherence can range in value from 0 to 1, where 0 indicates no correlation and 1 indicates perfect correlation; i.e. the phase and amplitude of one recording can be perfectly determined from another recording. The changes in coherence were then calculated as percentage changes with respect to the baseline, which we defined as the average coherence between 3 and 4 seconds prior to onset of muscle activity.

## Additional Information

**How to cite this article**: Talakoub, O. *et al*. Time-course of coherence in the human basal ganglia during voluntary movements. *Sci. Rep.* **6**, 34930; doi: 10.1038/srep34930 (2016).

## Supplementary Material

Supplementary Information
