# Supplementary material for: Time-course of coherence in the human basal ganglia during voluntary movements
Source: Sci Rep. 2016 Oct 11;6:34930. doi: 10.1038/srep34930 (PMC5057143; doi:10.1038/srep34930)
Supplement: Supplementary Information [file srep34930-s1.pdf]

# Time-course of coherence in the human basal ganglia during voluntary movements

**Omid Talakoub[1,2 *], Bogdan Neagu[3], Kaviraja Udupa[3], Eric Tsang[3], Robert Chen[3,4], Milos R Popovic[2,5], and Willy Wong[1,2 *]**

[1]Department of Electrical and Computer Engineering, University of Toronto, Canada
[2]Institute of Biomaterials and Biomedical Engineering, University of Toronto, Canada
[3]Toronto Western Research Institute, University Health Network, Canada
[4]Division of Neurology, Faculty of Medicine, University of Toronto, Canada
[5]Rehabilitation Engineering Laboratory, Toronto Rehabilitation Institute, University Health Network, Canada
[*]omid.talakoub@utoronto.ca, willy.wong@utoronto.ca

## Supplementary Table

| Age | Site | Predominant symptoms pre-operatively | Medication (dose/day, LED/day in PD) | Therapeutic DBS settings | DBS pair used in the analysis |
|---|---|---|---|---|---|
| 49 | GPi | Cervical dystonia, pain in neck and rest of body. | - | RGPi 2.7 V/60 $\mu$s/130 Hz/ (1-, C+); LGPi 2.6V/60 $\mu$s/130 Hz/ (5-, C+) | 2-1, 6-5 |
| 58 | GPi | Cervical dystonia, pain in neck | clonazepam 1.5 mg, gabapentin 1800 mg, nortriptyline 400 mg, lorazepam 3 mg, codeine | RGPi 2.9V/60$\mu$s/130 Hz/ (2-, C+); LGPi 4V/60$\mu$s/130Hz/ (5-, C+) | 2-1, 6-5 |
| 52 | GPi | Cervical dystoina with right laterocollis and retrocollis, pain | Clonazepam 2mg, citalopram 40 mg | RGPi 3.0V/60$\mu$s/130Hz/ (3-, C+); LGPi 2.9V/90$\mu$s/130Hz/ (6-, C+) | 2-3, 5-6 |
| 53 | GPi | Cervical dystoina with left torticollis | Lorazepam 12mg | LGPi 3V/60$\mu$s/130Hz/ (2-, C+); RGPi 1.8V/60$\mu$s/130Hz/ (6-, C+) | 2-1, 5-6 |
| 88 | GPi | Right hemidystonia | clonazepam 3 mg | LGPi 2.5V/60$\mu$s/60Hz/ (5-, C+) | |
| 45 | GPi | Dopa-responsive dystonia of the right leg, gait impairment | 1090 mg | RGPi 1.4V/60$\mu$s/130Hz (3-, C+); LGPi 4.0V/60$\mu$s/130Hz/ (7-, C+) | 2-3, 6-7 |
| 44 | GPi | Orofacial dystonia/Craniocerfical dystonia/Meige's syndrom + pain | Lorazepam 3mg | RGPi 4.3V/60$\mu$s/130Hz (1-2-, C+); LGPi 3.3V/120$\mu$s/130Hz (7-, C+) | 2-1, 6-7 |
| 54 | STN | Longer OFF periods, freezing | 1000 mg | RSTN 4.0V/60$\mu$s/80Hz, (3-, C+); LSTN 4.4V/60$\mu$s/80Hz/ (6-, C+) | 2-3, 5-6 |
| 65 | STN | PD | 600 mg | RSTN 3.4V/60$\mu$s/185Hz/ (2-, C+); LSTN 3.2V/60$\mu$s/185Hz/ (6-, C+) | 2-1, 5-6 |
| 50 | STN | Tremor dominant PD | 1200 mg | RSTN 3.8V/60$\mu$s/160 Hz/ (1-, C+); LSTN 2.8V/60$\mu$s/160 Hz/ (5-, C+) | 2-1, 5-6 |
| 50 | STN | PD | 1100 mg | RSTN 3.0V/60$\mu$s/130 Hz/ (2-, C+); LSTN 3.4V/60$\mu$s/130Hz/ (7-, C +) | 2-1, 6-7 |
| 56 | STN | Tremor dominant PD | 1800 mg | RSTN 2.8V/90$\mu$s/185Hz/ (2-, C+); LSTN 3.8V/90$\mu$s/185Hz/ (7-, C+) | 2-1, 6-7 |
| 44 | STN | Tremor dominant PD | 850 mg | RSTN 2.1V/60$\mu$s/130Hz/ (2-, C+); LSTN 2.3V/60$\mu$s/130Hz/ (5-, 6+) | 2-1, 6-5 |

**Table S1.** Clinical details of the patients studied.

## Supplementary Figures

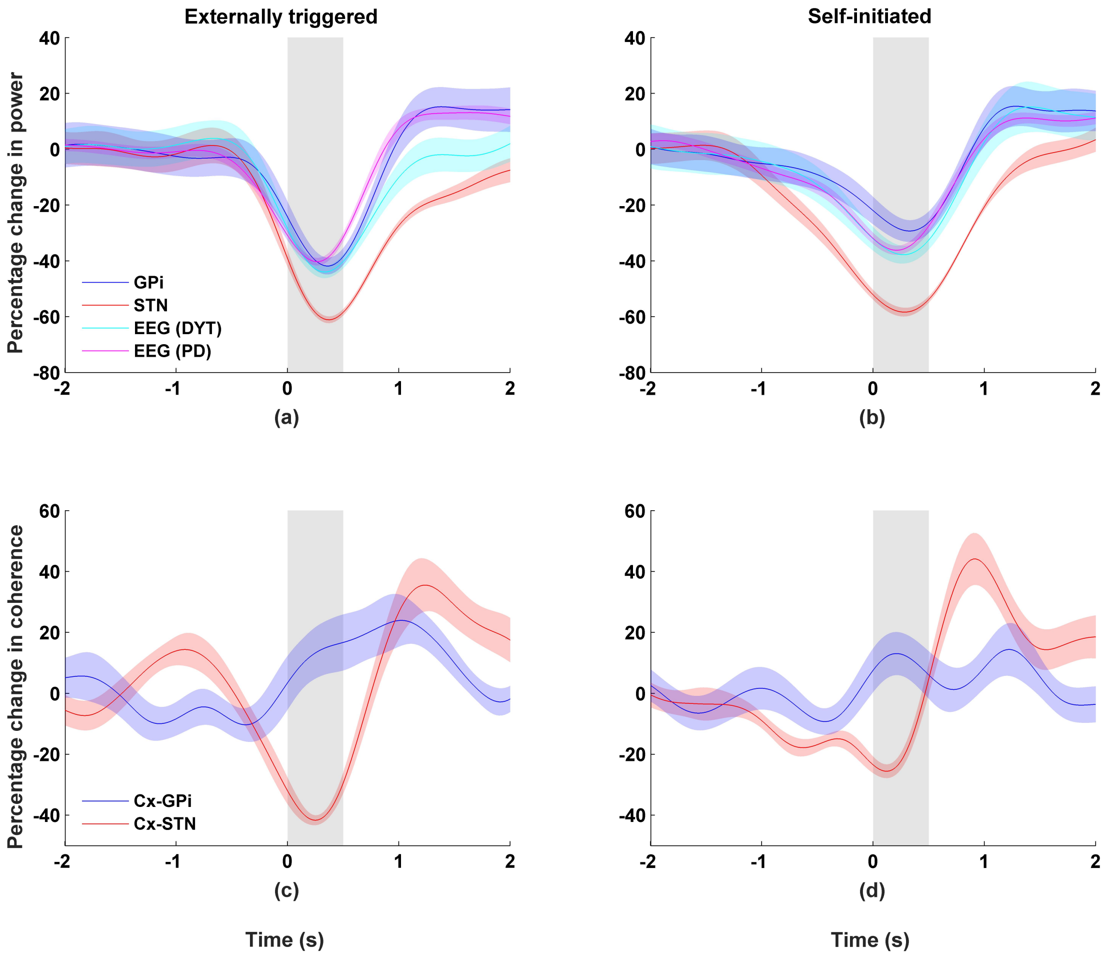


**Figure S1.** Beta band activity recorded from the side ipsilateral to the moving wrist. Movement duration indicated by grey area and shaded areas show standard errors. Left column indicates externally triggered task; right column the self-initiated task. Percentage changes for beta band (65-80Hz) showing (a/b) power (c/d) coherence between cortex-GPi or cortex-STN.

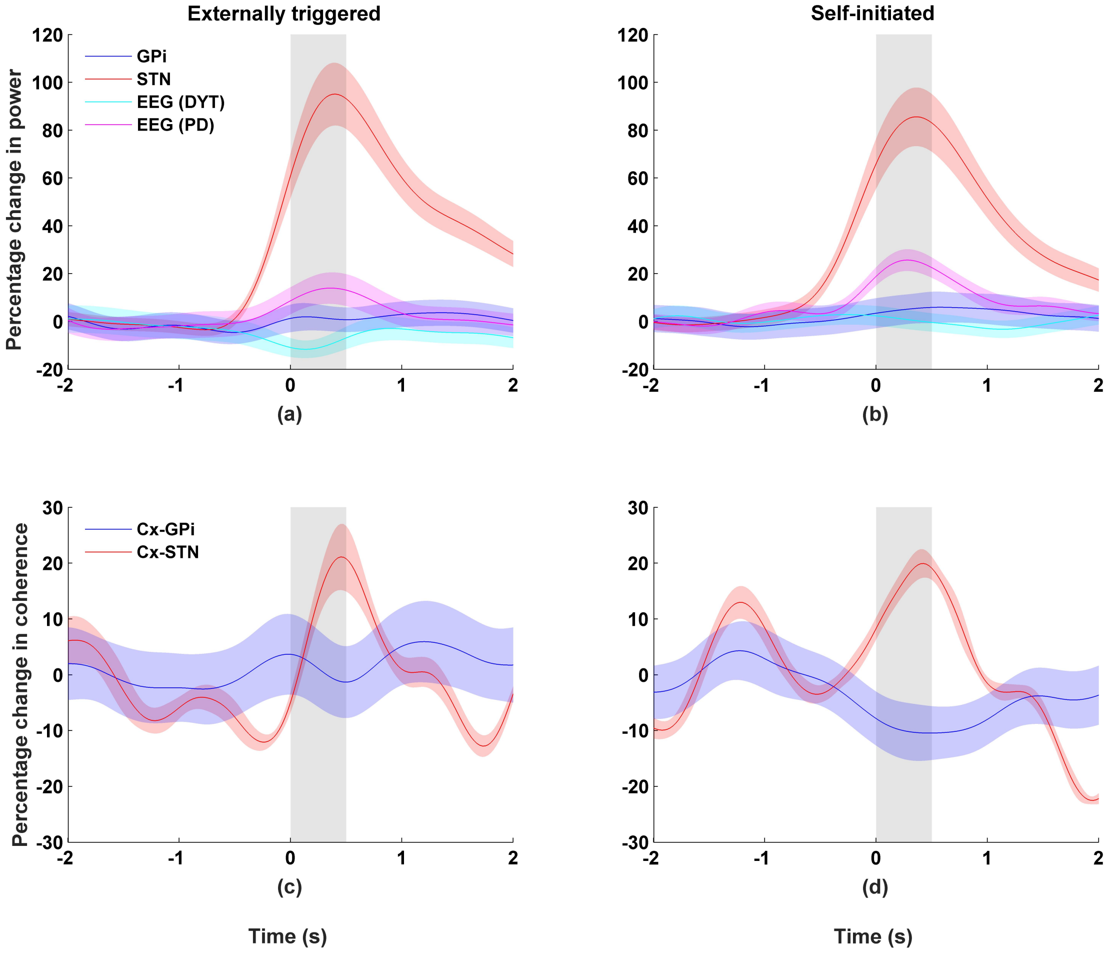


**Figure S2.** Gamma band activity recorded from the side ipsilateral to the moving wrist. Movement duration indicated by grey area and shaded areas show standard errors. Left column indicates externally triggered task; right column the self-initiated task. Percentage changes for beta band (65-80Hz) showing (a/b) power (c/d) coherence between cortex-GPi or cortex-STN.

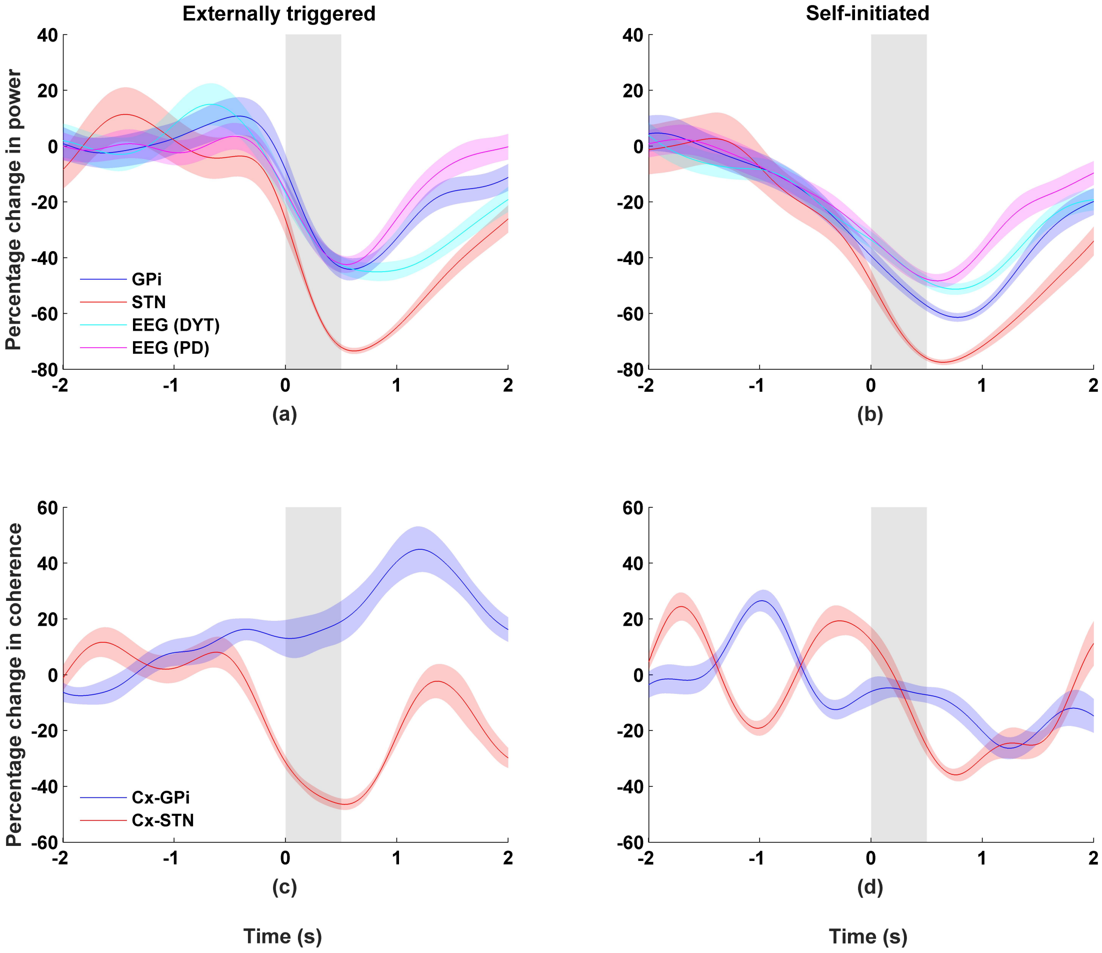


**Figure S3.** Alpha band activity recorded from the side ipsilateral to the moving wrist. Movement duration indicated by grey area and shaded areas show standard errors. Left column indicates externally triggered task; right column the self-initiated task. Percentage changes for beta band (8-12Hz) showing (a/b) power (c/d) coherence between cortex-GPi or cortex-STN.

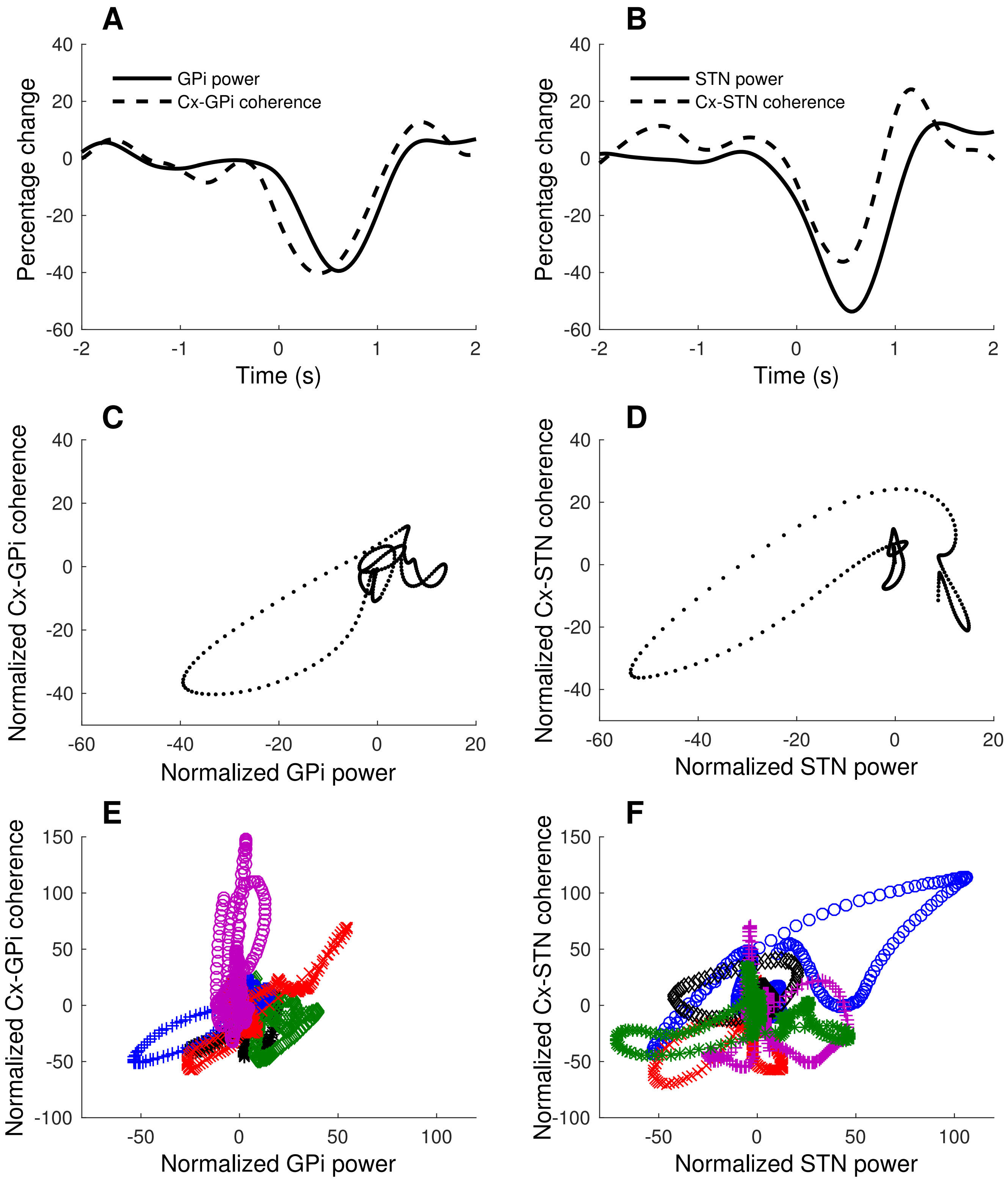


**Figure S4.** Percentage changes in power and coherence with respect to the baseline on the side contralateral to moving wrist during externally triggered movements. Baseline is defined as the average power between -4 and -3 seconds. (a and c) Grand average power and coherence for GPi patients; (b and d) for STN patients; (e) normalized coherence between Cx-GPi plotted against GPi power for five individuals; (f) normalized coherence between Cx-STN plotted against STN power for five individuals.
